# An Extremely Rare Presentation of Four Coronary Anomaly Patterns Originating from the Right Coronary Sinus

**DOI:** 10.1155/2022/7125401

**Published:** 2022-06-28

**Authors:** Nabil Braiteh, Wajeeh Rehman, Wajiha Ali, Owais Ahmed

**Affiliations:** ^1^United Health Services Hospitals, Wilson Medical Center, Department of Cardiology, 30 Harrison St, Johnson City, NY, USA; ^2^United Health Services Hospitals, Wilson Medical Center, Department of Internal Medicine, 33-57 Harrison St, Johnson City, NY, USA; ^3^American University of Antigua College of Medicine, USA; ^4^United Health Services Hospitals (Heart & Vascular Institute), Wilson Medical Center, Department of Cardiology, 30 Harrison St, Johnson City, NY 13790, USA

## Abstract

**Background:**

“Coronary anomaly” is defined as the coronary feature or pattern seen in <1% of the population. The most common CAAs are anomalies of origin, specifically having a separate LCX and LAD origin with an incidence of 0.41%. The second most common anomaly is the LCX arising from the RCA (0.37%). Treatment options include CABG, coronary unroofing, reimplantation, or medical therapy. *Case Presentation*. We present the case of an 85-year-old male who presents with an acute coronary syndrome who was found to have an extremely rare combination of different coronary anomaly patterns including left main coronary artery (LMCA) atresia, small LAD arising posteriorly from the right coronary cusp, anomalous left circumflex artery arising from the RCA, and an anomalous LAD arising from the left circumflex artery which is originating from the RCA.

**Conclusions:**

To the best of our knowledge, this is the first case report to describe four coronary anomalies in a single patient. When CAAs are diagnosed, it is of utmost importance for cardiologists to do further imaging and workup that might include a stress test to be able to offer patients the best management options.

## 1. Introduction

Is it a coronary anomaly or a normal variant? This has been a challenging question to answer in the literature and between experts. Villa et al. proposed to define “normal variants” as any coronary pattern that is observed in >1% in the general population, and a “coronary anomaly” is the coronary feature or pattern seen in <1% of the population [1].

Normal variants include coronary codominance, inferior wall supply, AV nodal supply, ramus intermedius, separate origin of the conus branch, myocardial bridging, acute takeoff of LCX, high take-off of the coronary ostium, and shepherd's crook RCA.

Coronary anomalies are classified based on anatomical features, and they include anomalies of the ostium, of origin, of anatomy, and of termination; congenital absence; and hypoplasia.

The most common CAAs are anomalies of origin, specifically having a separate LCX and LAD origin with an incidence of 0.41%. The second most common anomaly is the LCX arising from the RCA (0.37%) [1, 2].

In concept, if a coronary arises from the contralateral side, it has 4 possible paths: (A) interarterial (between the aorta and pulmonary artery), (B) transeptal (subpulmonic); (C) prepulmonic (anterior to the right ventricular outflow tract), and (D) retroaortic (posterior to the aortic root). The first (interarterial) is considered malignant and is at higher risk for SCD especially when it is associated with an intramural segment (proximal part of the artery is contained within the aortic wall), while the latter three (transseptal, retroaortic, and prepulmonic) are benign with low risk for adverse events [1, 2].

Herein, we report an 85-year-old man who presented with an acute coronary syndrome and heart failure, who was found to have an extremely rare combination of different coronary anomaly patterns.

## 2. Case Presentation

We present an 85-year-old Caucasian male who presented to the emergency department (ED) with complaints of dyspnea and a productive cough. His past medical history is significant for dyslipidemia, hypertension, diabetes mellitus type 2, chronic kidney stage 3b, obesity, and obstructive sleep apnea. He denied tobacco use but did report occasional alcohol use. His family history was unremarkable. At baseline, the patient was able to ambulate and perform daily activities without functional limitations.

Physical examination showed an ill-appearing elderly male, in mild respiratory distress. Vital signs on admission noted blood pressure 124/93 mmHg, heart rate 120 beats per minute, respiratory rate of 24 breaths per minute, and oxygen saturation of 91% on room air initially. The patient was alert and oriented. Heart sounds revealed normal S1 and S2, breath sounds were rhonchorous and diminished bilaterally with evidence of bilateral lower extremity edema. EKG revealed an accelerated ventricular rate of 131 beats per minute and idioventricular rhythm with a right bundle branch block. Anterior posterior chest radiograph revealed cardiomegaly with evidence of mild bilateral hilar congestion. Troponin I level was 0.171 ng/mL.

**Figure 1:**
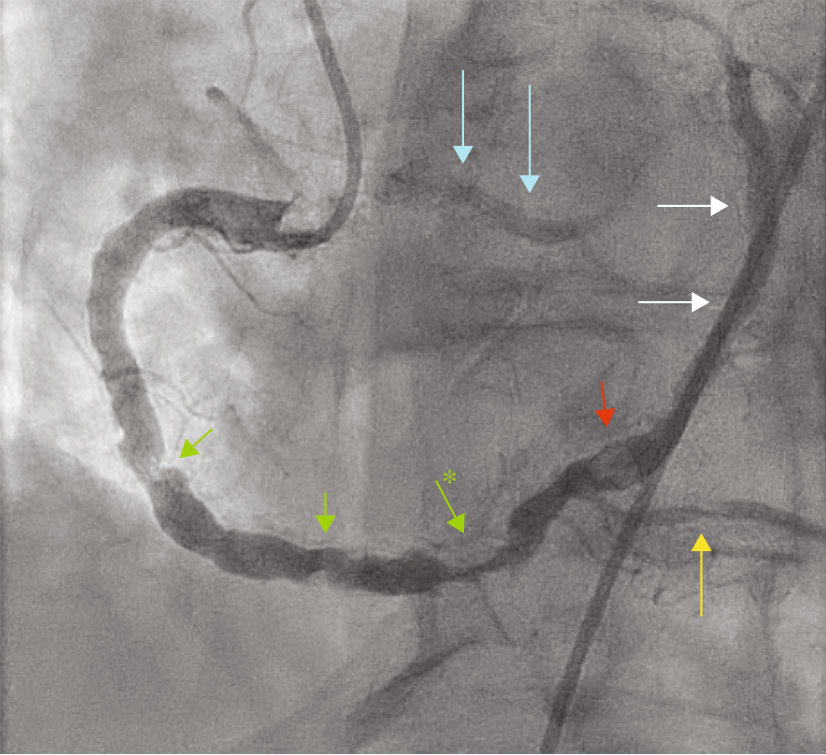
Right coronary angiogram (left anterior oblique 30°/cranial 30°) revealing a small anomalous LAD artery (blue arrows), a large ectatic RCA giving rise to the LCx (white arrows), multiple RCA lesions (green arrows) with an 80-90% mid to distal calcified eccentric lesion (green arrow with asterixis), a filling defect representing a thrombotic occlusion in the distal RCA (red arrow), and a 70% mid right posterior descending artery concentric lesion (yellow arrow).

**Figure 2:**
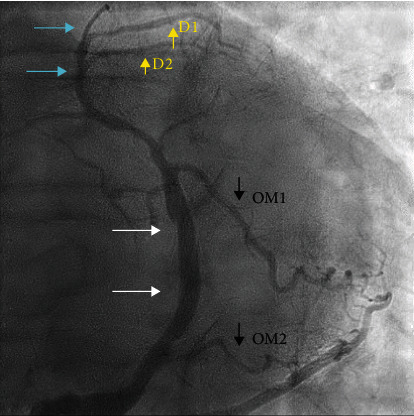
Right coronary angiogram (left anterior oblique 30°/cranial 30°) revealing an anomalous LCx artery (white arrows), the second small LAD artery (blue arrows), 1st and second diagonal arteries (yellow arrows), and 1st and second obtuse marginal arteries (blue arrows).

The patient was diagnosed with mild decompensated heart failure in the setting of non-ST elevated myocardial infarction. Oral clopidogrel 300 mg was administered along with heparin drip per ACS protocol. He was then transferred to the cardiovascular intensive care unit for close monitoring. Transthoracic echocardiogram revealed severely increased left ventricular thickness, normal left ventricular size with ejection fraction of 40-45%, and no wall regional wall motion abnormalities or significant valvular disease. After effective diuresis, the patient underwent cardiac catheterization which revealed anomalous coronary anatomy. The patient had no left coronary system and a large ectatic right coronary artery (RCA) with evidence of 2 left anterior descending arteries. The first was originating posteriorly from the right cusp (Figure 1, blue arrows) and the second (Figure 2, blue arrows) was originating from the anomalous left circumflex artery (Figures 1 and 2, white arrows). Additionally, he had an extremely ectatic large caliber right coronary artery (RCA) with multiple lesions (Figure 1, green arrows). The most significant was a 80-90% mid to distal calcified eccentric lesion (Figure 1, green arrow with asterixis). There was also evidence of a filling defect representing a thrombotic occlusion in the distal RCA (Figure 1, red arrow), and this gives rise to the anomalous left circumflex artery (LCX) (Figures 1 and 2, white arrows).

**Figure 3:**
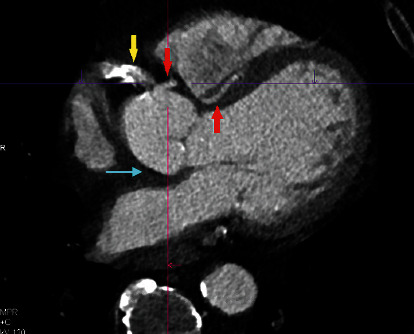
Coronary CT showed the absence of a left coronary system (blue arrow), a large calcified RCA originating posteriorly from the right coronary cusp (yellow arrow), and a small LAD artery originating from the right coronary cusp into the interventricular groove (red arrows).

**Figure 4:**
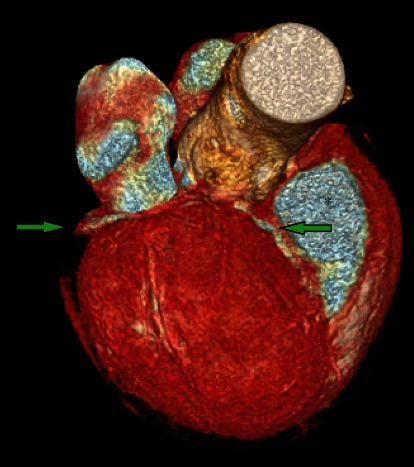
CT coronary showing a large calcified RCA originating posteriorly from the right coronary cusp (green arrow) and a small LAD artery originating from the right coronary cusp into the interventricular groove (green arrow with asterixis).

The above findings were confirmed by CT coronary, where it shows the absence of a left coronary system (Figure 3, blue arrow), large calcified RCA originating posteriorly from the right coronary cusp (Figure 3, yellow arrow, Figure 4; green arrow), and a small LAD artery originating from the right coronary cusp and into the interventricular groove (Figure 3, red arrows; Figure 4, green arrow with asterixis) which is also represented in Figure 1 by the blue arrows.

The patient had an episode of asymptomatic nonsustained idioventricular tachycardia followed by supraventricular tachycardia. As such, CT heart and coronary artery angiogram was done which confirmed the left coronary artery to arise from the aortic cusp making its course in front of the right ventricle between the great vessels thereby giving rise to a small caliber LAD. Likewise, the patient's circumflex artery appeared to also arise from the right coronary artery.

CABG was offered to the patient; unfortunately, he declined the intervention. He was medically optimized and discharged to rehab due to deconditioning. Three months after discharge, the patient suffered a cardiac arrest and passed after 25 minutes of unsuccessful CPR.

## 3. Discussion

Incidence of coronary arterial anomalies (CAAs) has been reported to range from 0.78% to 1.3% by invasive angiography and 0.99%-5.8% by coronary computed tomographic (CT) imaging [3, 4]. The wide range of incidence is likely due to the variability in defining “normal variants” and “anomalous” arteries. The most common CAA is having a separate LCX and LAD origins with an incidence of 0.41%. The second most common anomaly is the LCX arising from the RCA (0.37%) [1, 2].

Most CAAs do not have clinical significance or complications, but some (transseptal and interarterial) anomalies can lead to significant manifestations including heart failure, myocardial infarction, angina, and arrhythmias [5]. The pathophysiology is not completely understood, but several mechanisms have been proposed that include interarterial coronaries coursing intramurally through the wall of the aorta resulting in their compression due to aortic pulsation, angulation of the anomalous vessel acting like a valve-like ostial ridge, spasm of the anomalous artery due to ischemia or endothelial injury caused by its long distance, acute angle of take-off that can kink or occlude during exercise causing coronary hypoperfusion, and the fact that an artery has abnormal origin and take-off makes it more prone for atherosclerosis [1, 3, 6].

Spasm of the anomalous artery due to ischemia or endothelial injury is caused by its long distance, acute angle of take-off that can kink or occlude during exercise, and the fact that an artery has abnormal origin and take-off makes it more prone for atherosclerosis[1, 3, 6]CAAs are the second most common cause of sudden cardiac death accounting for 17% after hypertrophic cardiomyopathy (36%) reported by the U.S national registry of competitive athletes [7].

Diagnosis of CAAs can be done by noninvasive imaging techniques such as echocardiography, coronary CT angiography, and MR imaging. Alternatively, they can also be diagnosed invasively by coronary angiography.

While echocardiography has a limited role in adults, it has a good diagnostic benefit in children and adolescents (0-21 years of age) [7] Previously, coronary angiography was the standard test to diagnose CAAs, but with advancement in imaging, ECG-gated coronary CT angiography was shown to be more sensitive in multiple studies [8, 9]. While MRI has less radiation exposure, it has a similar diagnostic accuracy in comparison with CT [4].

Treatment of CAAs is complex and can be challenging specifically with the anomalous origin of the coronary artery from the opposite sinus (ACAOS). Decision to treat is mainly made for ACAOS with an interarterial course. While some experts recommend treatment for left ACAOS for asymptomatic patients, others take into account results of stress testing and age to decide on treatment. Ischemic symptoms will definitely warrant treatment. Options include CABG, coronary unroofing, reimplantation, or medical therapy [1, 4].

Our patient had four coronary anomalies detected: left main coronary artery (LMCA) atresia, small LAD arising posteriorly from the right coronary cusp, anomalous left circumflex artery arising from the RCA, and an anomalous LAD arising from the left circumflex artery which is originating from the RCA (Figures 1–4). To the best of our knowledge, this is the first case report to describe the above combination of coronary anomalies in a single patient.

It was an interesting finding to see a late presentation in a patient with the above 4 anomalies described. Survival until the age of 85 is most likely explained by the presence of large caliber RCA to supply the whole right and left side and the absence of an interarterial course. Another explanation is that he was more sedentary and was never involved in competitive sports. The Italian cardiological guidelines for competitive sport eligibility in athletes with heart disease [10] do recommend further workup that may include a CTA in symptomatic athletes. Had he been involved in sports, his coronary anomaly could have been diagnosed at an earlier time and could have been corrected with surgery.

## 4. Conclusion

Although CAA is a rare entity, when diagnosed, it is of utmost importance for cardiologists to do further imaging in order to differentiate between benign and high-risk anomalies. Moreover, a heart team approach and further workup might be required, including a stress test in order to offer patients the best management options.
